# Changes in Tubular PVDF Membrane Performance During Initial Period of Pilot Plant Operation

**DOI:** 10.3390/membranes15040119

**Published:** 2025-04-09

**Authors:** Marek Gryta, Wirginia Tomczak

**Affiliations:** 1 Faculty of Chemical Technology and Engineering, West Pomeranian University of Technology in Szczecin, Piastów Ave. 17, 70-310 Szczecin, Poland; 2 Faculty of Chemical Technology and Engineering, Bydgoszcz University of Science and Technology, 3 Seminaryjna Street, 85-326 Bydgoszcz, Poland; tomczak.wirginia@gmail.com

**Keywords:** ultrafiltration, pilot plant, tubular PVDF membrane, membrane compression, fouling

## Abstract

Ultrafiltration (UF) is increasingly used in the food industry and for wastewater treatment and water reuse. Knowledge of the membrane properties that stabilise during the initial period of module operation in an industrial plant is essential for design purposes. This paper presents the experimental tests carried out using a pilot plant with an industrial PCI B1 membrane module. The module was equipped with tubular FP100 (100 kDa) polyvinylidene fluoride (PVDF) membranes used to separate carwash wastewater. The effect of membrane compaction during the first few days of the process on changes in permeate flux and dextran (40–500 kDa) separation rate was investigated. The effect of fouling, membrane washing with P3 Ultrasill 11 solution (pH = 12) and maintenance with sodium metabisulfite solution on the stabilisation of the technological performance of the plant was determined.

## 1. Introduction

Ultrafiltration (UF) is used to remove turbidity, particles and microbial pathogens from treated water. The development of membrane cleaning processes such as backwashing, chemical cleaning and pre-treatment has led to the increasing use of UF for the treatment of high-turbidity water and wastewater [1,2,3,4] and for separation in the food processing industry [5,6].

The main problem limiting industrial applications of UF is fouling [7,8], which is reduced by using different methods of membrane rinsing and washing [1,3,4,9]. The intensity of fouling can also be reduced by modifying membrane properties, such as increasing hydrophilicity [4,10], which has been shown to reduce the fouling of polyvinylidene fluoride (PVDF) membranes [11].

Membrane fouling can be reduced by applying appropriate operating conditions to the modules. Their manufacturers usually specify a minimum feed flow rate, which, by increasing the shear force at the membrane surface, can remove excess sediment from the membrane surface and reduce gel/cake formation [3,5,12]. The applied transmembrane pressure (TMP) is also of great importance [13]. For wastewater treatment, TMP values not exceeding 0.2 MPa are generally used to reduce sediment accumulation on the membrane surface and to reduce its compressibility [2,3,6,14,15]. Hydraulic pressure also causes membrane compression, which affects membrane performance [9,16].

It has been widely documented that the fouling phenomenon may lead to a change in the membrane properties during the long-term process, especially with cyclic membrane washing [3,14,17]. For chemical cleaning, sodium hydroxide (NaOH) solutions with various additives (e.g., surfactants), sodium hypochlorite (NaClO) and acids have been used [3,10,12,15,18,19]. These cleaning agents are chemically aggressive and can alter the structure of polymeric membranes, affecting their performance [8,17,20]. It is therefore difficult to assess the performance of full-scale UF systems from short laboratory tests using small-area modules. Therefore, in order to obtain the necessary data for the design of industrial plants, it is important to carry out pilot tests using industrial modules, as presented in this work.

Protein, fat and oil are common foulants in wastewater separation [5,10,18]. Alkaline cleaning agents such as P3 Ultrasil 11 have been successfully used to remove such contaminants [21,22]. In addition, wastewater often contains various chemicals that can damage polymer membranes. Carwash wastewaters have such properties; however, the PVDF tubular membranes have been successfully used for their separation [23]. Carwash wastewater caused significant fouling, which was reduced by washing the membranes with alkaline solutions (pH = 11–12) [24]. In this case, UF laboratory tests performed for 100–200 h showed good chemical resistance of the membranes tested [23,25]. However, the cleaning solutions used contained NaOH, which can cause degradation of polymeric membranes. Therefore, much longer pilot-scale tests are required for industrial applications.

Undoubtedly, publications presenting the results of UF pilot plant studies are scarce [1,2,3,18]. They mostly describe fouling-induced permeate flux decline and the effectiveness of the various membrane cleaning methods [3,10,14,15]. Cleaning-in-Place (CIP) systems are generally used to clean the modules in UF plants [1], but this creates additional problems in the treatment with the resulting rinsing effluents [19,26].

In industrial plants, despite cyclical membrane cleaning, the membrane generally fails to remove fouling completely, resulting in reduced initial permeate flux. Instead, these residual contaminants penetrate into the largest pores, which can increase rejection [18,23]. As a result of these phenomena, membrane properties change and then, stabilise during the initial period of module operation. This paper shows the impact of the applied TMP values and the chemical membrane cleaning on the changes in permeate flux and separation properties of PVDF tubular membranes during the first weeks of operation.

## 2. Materials and Methods

### 2.1. UF Studies

**Figure 1:**
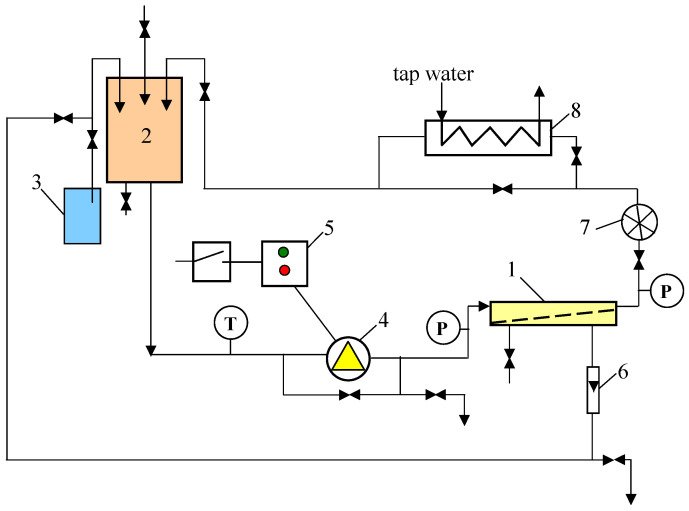
UF pilot plant. 1—tubular module, 2—feed tank, 3—permeate tank, 4—pump, 5—electric supply, 6—rotameter, 7—flowmeter, 8—heat exchanger, T—thermometer, P—manometer.

The UF process was investigated using a PCI B1 tubular module mounted in a pilot plant, the design of which is presented in [27] and schematically shown in Figure 1.

The B1 module was equipped with 18 tubes of FP100 membrane (diameter, length and total membrane area were 1.25/1.27, 1.2 m and 0.9 m^2^, respectively). A parallel flow head was installed in module B1, allowing similar process conditions in each FP100 tube. The FP100 membranes (100 kDa) are made of polyvinylidene fluoride (PVDF) and are manufactured by PCI Membranes (Kostrzyn Wielkopolski, Poland). The UF studies were performed at transmembrane pressure (TMP) in the range of 0.05 to 0.4 MPa (0.5–4 bar). The feed was pressurised by a centrifugal pump (type VNR-8, Grundfos, Denmark) and flowed inside the FP100 tubes. Hydraulic pressure affects the performance of the centrifugal pumps; therefore, the feed flow velocity was dependent on TMP and was 1.9 m/s (TMP = 0.1 MPa) and decreased to 1.3 m/s at TMP = 0.4 MPa. The UF installation was cooled with tap water, and the feed temperature was 298 ± 1 K.

Tap water softened by nanofiltration (NF) (permeate conductivity 50–60 µS/cm) was used to prepare the feed. Ion chromatographic analysis showed that the NF permeate contained [mg/L]: 2.25 Na^+^, 0.51 Ca^2+^, 0.31 K^+^, 0.08 Mg^2+^, 2.11 Cl^−^, 0.25 SO_4_^2−^, and 0.15 NO_3_^−^. During the UF tests, the feed tank was filled with 100–150 L of NF permeate. Tests were conducted at constant feed concentrations, with the permeate and retentate returned to the tank. The UF tests were carried out at constant feed flow, and the change in permeate flow was measured.

At the end of the studies, the UF process was carried out using synthetic carwash wastewater consisting of a mixture of 0.5%/*v*/*v* foaming agent solution and 0.2%/*v*/*v* Hydrowax solution (EuroEcol, Łódz, Poland). This mixture composition corresponds to the concentrations of effluents generated at car washes and has been used in previous laboratory studies of UF carwash wastewater [25]. The mixture used contained anionic (460 mg/L) and non-ionic (23 mg/L) surfactants in addition to waxes, and the chemical oxygen demand value was 2560 mg/L. The exact chemical composition of the agents used to prepare the test mixture (foaming agent and hydrowax) has been reported in publications [24,25].

After completion of a given series of UF tests, the installation was rinsed twice with water (NF permeate). Chemical cleaning was also performed periodically for 30 min. For washing the tested membranes, the PCI manufacturer recommended the cleaning agent P3 Ultrasil 11 containing NaOH and detergents (Suturamed, Szczecin, Poland). A 0.1 wt.% solution (pH = 12) was used to wash the membranes. Prior to a shutdown for several days for membrane maintenance, the unit was rinsed with a 0.25 wt.% sodium metabisulfite (Na_2_S_2_O_5_) solution (ChemLand, Stargard, Poland).

During the UF process, membrane separation properties can change. Changes in the membranes’ permeability were assessed by periodically measuring the dependence of permeate flux on TMP. For this purpose, the pressure was increased to TMP = 0.4 MPa and the permeate flux value was noted after 5 min, then the pressure was decreased stepwise by 0.05 MPa, and the next flux value was noted after 1 min (rotameter, ±2 L/h).

### 2.2. Analytical Procedures

Chemical cleaning can change the structure of the membranes, affecting the separation. To analyse this phenomenon, the UF of standard solutions of dextran with different molecular weights in the range of 40 and 500 kDa (Polfa, Łódź, Poland) were carried out. Separation studies were performed at TMP = 0.1 MPa. After rinsing, the UF plant was filled with a new batch of NF permeate (100 L), and the UF process was started. In order to thoroughly clean the permeate side (volume 8 L), the B1 module housing was emptied of permeate twice. Dextran was then added to the feed tank (5 g/100 L), and after 5 min, the permeate was removed from the module housing. After a further 20 min of UF, samples of the feed and permeate were taken for analysis. The dextran solution was then removed, the UF plant was rinsed, and the cleaning process was repeated to prepare the next dextran solution for UF separation. Additionally, the separation of dextran solutions with concentration of 1 g/L was also carried out.

The concentration of dextran in both feed and permeate was determined comparatively by the determination of total organic carbon (TOC). TOC and inorganic carbon (IC) values were analysed using a “Multi N/C 3100” analyser (Analytik Jena, Germany). The rejection efficiency R [%] was determined as follows:

(1) $$R=\frac{C_{F}-C_{P}}{C_{F}}100\%$$

where C_P_ [mg/L] and C_F_ [mg/L] are the measured concentrations of the permeate and feed, respectively.

The membrane morphology was studied using an SU8020 (Hitachi High Technologies Co., Tokyo, Japan) scanning electron microscope (SEM).

The Hach cuvette tests were used to determine the concentration of surfactants (LCK 333—nonionic, LCK 432—anionic) and chemical oxygen demand—COD (LCK 1014). The ion composition of solutions was analysed using an 850 Professional IC ion chromatograph (Herisau Metrohm AG, Herisau, Switzerland).

## 3. Results and Discussion

### 3.1. Active Layer Porosity

**Figure 2:**
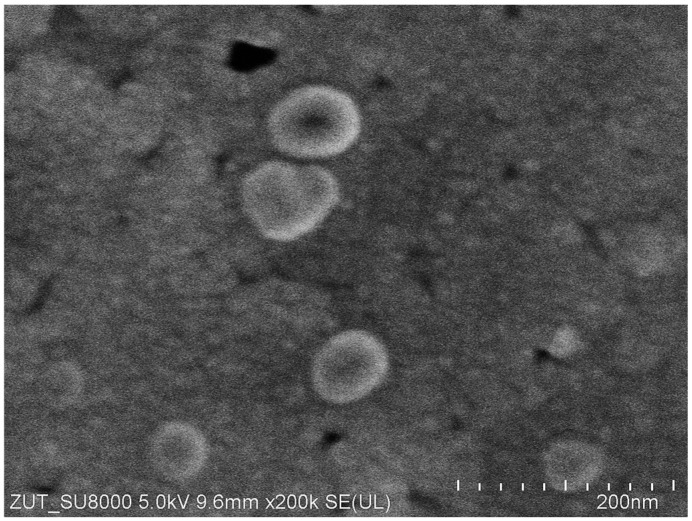
SEM image of new FP100 membrane surface.

The separation properties of the FP100 membranes are determined by the active layer (thickness of less than 1 μm), which is formed on a wide, porous support layer with a thickness of more than 100 μm [23,27]. UF membranes have fine pores; however, SEM observations have shown that large pores of 20–40 nm are also present on some parts of the membrane surface (Figure 2). The presence of these pores results in a high permeate flux, above 400 LHM, during the initial UF period [23]. However, this value decreases very quickly due to the blocking of these pores by suspended particles in the feed [7], which are difficult to remove even when the pilot plant is fed with pure water [27].

The properties of the membranes change once the UF process starts, not only due to the blocking of the pores as shown in Figure 2, but also due to the compression of the membranes. Therefore, the correct choice of plant conditions has a significant impact on the subsequent performance of the membranes [9].

### 3.2. Membrane Compression

**Figure 3:**
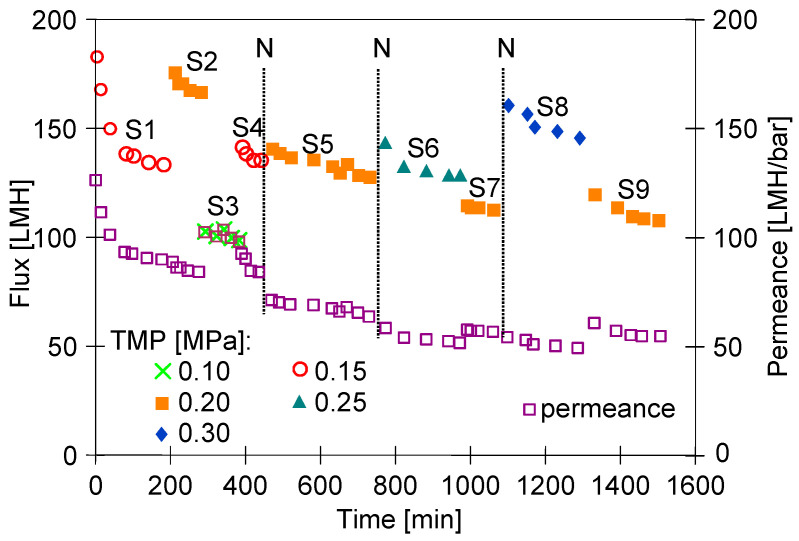
Changes in permeate flux and permeance as a function of TMP and membrane stabilisation time. N—night break. NF permeate used as a feed.

Due to the compressibility, the membrane permeability changes under the influence of the TMP and usually stabilises after a few changes in the TMP [17,28,29]. When starting a new membrane module, the process usually starts with lower TMP values, which are gradually increased. After rinsing out the preservatives at TMP = 0.15 MPa, the membranes tested showed a high permeate flux close to 200 LMH, which decreased to 135 LMH after 3 h of water filtration (Figure 3). Two further UF series were carried out for TMP = 0.2 and 0.1 MPa. The S4 series was then returned to TMP = 0.15 MPa and a flux of 135 LMH was obtained again, which could indicate a stabilisation of membrane performance. The permeate flux values obtained were similar to those achieved for PVDF membranes in another paper [10]. In the tests carried out, the feed was NF permeate, the composition of which does not cause fouling of the UF membranes. However, in addition to the feed, the construction material of the pilot plant can also generate micropollutants [27]. Such constituents can be rapidly deposited in the large pores shown in Figure 2. This phenomenon, in addition to membrane compaction, could be the reason for the rapid decrease in flux observed during the S1 series.

The results presented in the current study show that any change in TMP affects membrane permeability. The largest changes in permeate flux were observed during the first hour of module operation (Figure 3, series S1), confirming the results presented in [16]. However, flux changes also occurred during the subsequent hours of the UF process, indicating that much longer operating times than 60 min are required to stabilise the initial permeate flux. At the end of the S4 series run, the plant was shut down, and the water was left in the plant.

After an interruption of 14 h, the filtration process was restarted at TMP = 0.2 MPa and 140 LMH was obtained (series S5), which is lower than the value obtained for this TMP during the S2 series (165 LMH). This finding indicates that interruptions also have an effect on membrane performance. The pilot plant was operated in a laboratory and, for safety reasons, the UF tests were carried out with a night break (about 12 h). However, such interruptions may also occur in small manual carwashes, which generally do not operate at night. When operating the plant, it should be taken into account that the membranes are more difficult to wash after a longer break [14].

On subsequent days of the UF process, the results obtained for TMP = 0.2 MPa already showed a much smaller decrease in flux, which stabilised at 110 LMH (series S9). This indicates that, in order to stabilise the membranes, an initial start-up of the plant should be carried out for at least 2–3 days. If one were to take the value obtained during series S2 instead of the permeate flux from series S9, the analysis of the flux changes occurring during further UF tests would be overstated.

A membrane can be considered stabilised if, despite changes in TMP, the membrane permeance remains similar. Analysis of the changes in permeance showed that after two days of UF testing (from series S6), changes in TMP no longer caused significant changes in permeance, which stabilised at 55 LMH/bar (Figure 3). As a result, after increasing the TMP from 0.2 (S7) to 0.3 MPa (S8) and then reducing it back to 0.2 MPa (S9 series), a permeate flux similar to that of the S7 series was obtained.

**Figure 4:**
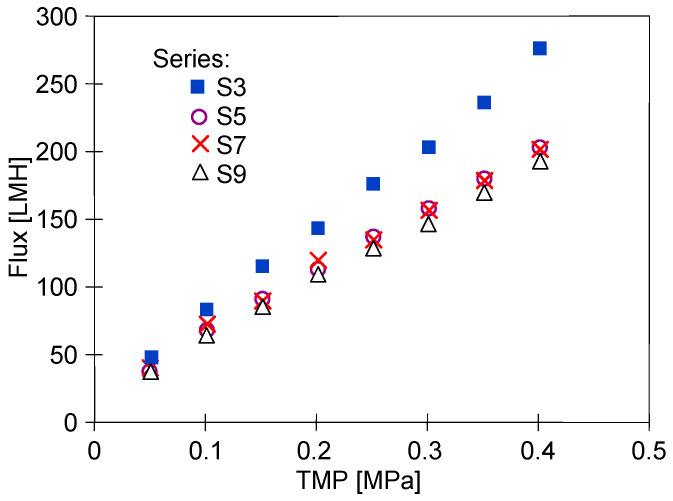
Changes in the permeate flux during UF tests. Measurements series taken after the UF tests series shown in Figure 3.

The conclusion that the membranes stabilised after three days of testing is also supported by the study of the effect of TMP on flux changes carried out at the end of a given measurement series (Figure 4). Module performance was highest on the first day of operation and then stabilised at a lower level on subsequent days. This suggests that the membranes were still unstable after 6 h of operation (series S3) and that values obtained over a much longer period of module operation should be used to assess changes in membrane permeability.

The results presented above indicate that it takes several days for the operating parameters of the new UF module to stabilise. As shown in other pilot studies, this period also depends on the properties of the feedstock, and in the case of wastewater, stable plant operation was only achieved after 15 days [15].

### 3.3. Dextran Separation

Initial changes in the membrane parameters can affect not only the flux but also the retention degree. Therefore, after the permeate flux had stabilised, the separation tests of standard solutions of dextran were carried out (Table 1, series D1). The rejection values obtained differed significantly from the MWCO declared by the manufacturer (100 kDa). Similar discrepancies for clean membranes have been reported in other work [30].

**Figure 5:**
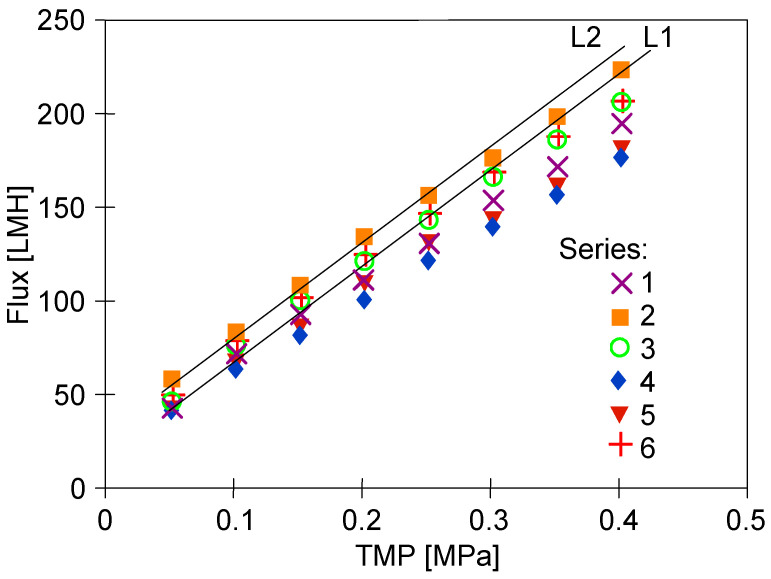
Changes in the permeate flux during module cleaning performed after separation of diluted dextran solutions. Series: ‘1’—flux after series D1 dextran separation, ‘2’—module washed with P3 Ultrasill 11, ‘3’—module rinsed with water, ‘4’—water flux after D2 dextran separation series, ‘5’—rinsing with water, ‘6’—module washed with P3 Ultrasill 11. L1, L2—line.

After dextran separation and rinsing with water, the flux values (Figure 5, series ‘1’) were similar to those obtained after the S9 series (Figure 3). However, at higher TMP values, the permeate flux shows a deviation from a linear increase (line L1), indicating membrane fouling [29]. In the absence of fouling, the water flux for a membrane is linearly proportional to the TMP applied. The flux values increased after washing the system with P3 Ultrasil 11 solution and rinsing with water (Figure 5, series ‘2’). This was because the alkaline solution (pH = 12) removed residual dextrans from the membranes, as evidenced by the improved linearity of flux growth (Figure 5, line L2). In addition, the chemical cleaning also removed some of the sediment from the large pores shown in Figure 2, which increased membrane permeability [23].

When the feed was replaced with a new batch of water, and after 30 min rinsing the installation, the flux decreased (Figure 5, series ‘3’). This confirms that such pores are rapidly re-clogged by micro-pollutants generated by the construction material of the pilot plant, which will also be present if distilled water is used as a feed [27].

Due to the fouling phenomenon, membranes are washed cyclically during UF tests. The cleaning agents can change the membranes’ structure, which alters their separation properties [8,17,20]. The experimental results reported above show that determining the performance of the resulting membranes in industrial applications is of great importance.

The used cleaning agent P3 Ultrasil 11 contains NaOH, which alters the polymeric structure of the membranes and consequently increases their permeability [24,31]. Therefore, the dextran separation tests were repeated after chemical cleaning. The retention values obtained were slightly lower (Table 1, ‘D2 series’). However, a previous study showed that repeated alkaline washing of the PVDF membranes did not significantly affect the separation of wastewater collected from the touchless car washes [23].

**Figure 6:**
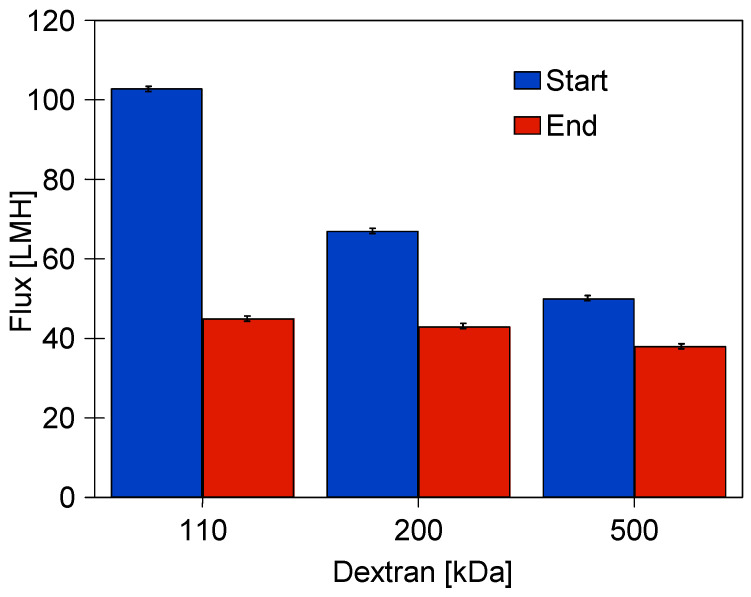
Changes in permeate flux during the separation of dextran solutions (1 g/L). TMP = 0.1 MPa. UF process time: Start—5 min, End—30 min.

However, re-running the system with dextran solutions (series D2) increased fouling. After flushing the system with water, the decrease in flux (Figure 5, series ‘4’) was greater than after the previous D1 series of dextran separation (series ‘1’). Subsequent rinsing of the system with a new batch of water increased the flux only slightly (series ‘5’). After washing with P3 Ultrasill 11 solution (series ‘6’), a performance similar to the initial one was restored, but the flux was lower than after the first membrane washing (Figure 5, series ‘2’). This result indicates that during UF, not only a large amount of feed components accumulates on the membrane surface, but also internal fouling develops [3]. The effect that larger pores, as shown in Figure 2, are more susceptible to fouling is well documented [7]. Dextrans are water soluble, but during UF, they can adsorb onto membranes and form a gel layer. In order to investigate these phenomena in the next run, the dextran concentration was increased to 1 g/L. This increased the retention of the 500 kDa dextran to 94% but significantly reduced the permeate flux (Figure 6).

**Figure 7:**
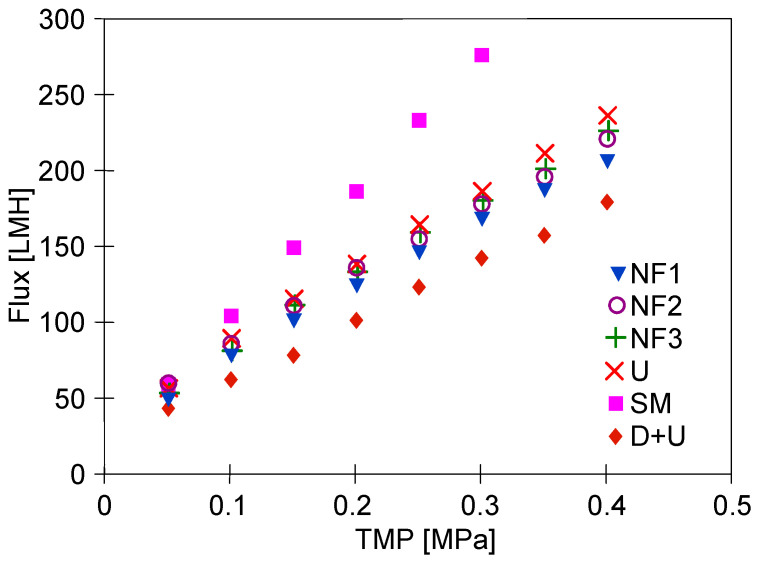
Changes in the permeate flux during module cleaning performed after separation of dextran solutions (1 g/L). Points: D+U—after dextran separation membranes washed with P3 Ultrasill 11 and water, U—second washing with P3 Ultrasill 11, NF1, NF2, NF3—membranes rinsed with NF permeate, SM—membranes rinsed with 0.25% sodium metabisulfite solution.

Increasing the concentration of dextran in the feed not only reduced the flux but also worsened the efficiency of membrane washing. After the first washing of the system with the P3 Ultrasil 11 solution, the flux for TMP = 0.4 MPa only increased to 178 LMH, whereas before, it was 205 LMH (Figure 5, series ‘6’). Repeating the washing with the P3 Ultrasil 11 solution increased the flux to 225 LMH (Figure 7, points ‘U’). The flux value decreased slightly after the module was rinsed with NF permeate (points ‘NF1’).

It must be mentioned that during prolonged plant shutdowns, the membranes must be maintained to prevent bacterial growth. Therefore, prior to shutdown, the plant was flushed (30 min) with 0.25% sodium metabisulfite solution, and the plant was drained. After 7 days of shutdown, the plant was rinsed twice with water, and a significant increase in permeate flux was observed (Figure 7, SM points). Presumably, the preservative also cleaned the large pores shown in Figure 2. Water filtration was then carried out for 5 h (TMP = 0.1 MPa). After this period, the capacity of the plant decreased (Figure 7, points ‘NF2’). After an overnight break, the unit was filled with a new batch of water, and after 2 h of rinsing, the permeate flux was almost unchanged (points ‘NF3’). Such a high value (e.g., 220 LMH for TMP = 0.4 MPa) indicates that the membranes were cleaned after preservation to a similar extent as after washing after the first series of dextran separations (Figure 5, series ‘2’). This finding demonstrates that the performed initial UF tests and repeated membrane washes resulted in a relatively stable level of module performance.

Fouling significantly reduces the membrane performance and consequently increases the cost of ultrafiltration [5]. It is suggested that its negative impact can be eliminated by the application of feed pre-treatment [4]. However, in small carwashes, it is not cost-effective to use extensive wastewater treatment systems. Hence, it is only possible to consider washing membranes with the detergent used for cars and the equipment that makes up the carwash, such as pumps and electronic flow controllers. Such a process solution has been presented in previous works [23,25]. Surfactants are used to wash membranes, but their presence in the feed also affects the properties of UF membranes [32]. However, studying the effect of detergents requires that the changes in membrane performance caused by installation rinsing and chemical washing have already been stabilised.

### 3.4. Wastewater Separation

**Figure 8:**
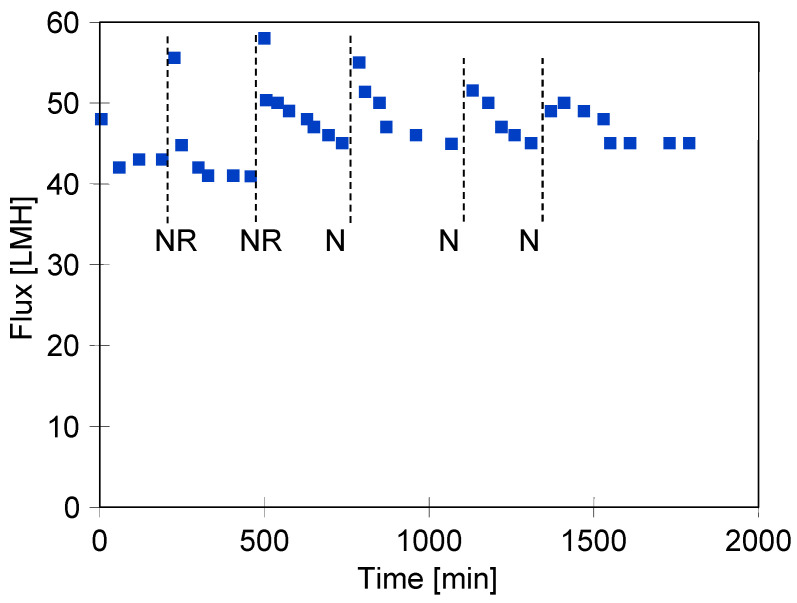
Changes in the permeate flux during the separation of synthetic carwash wastewater (0.5 vol% Foam cleaning agent + 0.2 vol.% Hydrowax). TMP = 0.1 MPa. NR—before night break installation rinsed with NF permeat, N—night break, wastewater remained in the plant.

The final series of studies tested the separation of model car wash effluent containing hydrophobic waxes in addition to surfactants. In the first two days, the wastewater was removed and the UF plant was rinsed with water before being stopped overnight. However, on the following days, the plant remained filled with the test wastewater. Rinsing with water increased the initial flux value, but this quickly decreased to a level of 45 LMH (Figure 8). This value was also obtained when the wastewater was kept in the plant overnight after the UF process. Car wash effluent contains the contaminants removed from the car, but still contains components that have detergent properties. Therefore, during the overnight standstill, due to their interaction and the expansion of the membrane (TMP = 0), the permeate flux also increased after the UF process was restarted before stabilising at a lower level.

**Figure 9:**
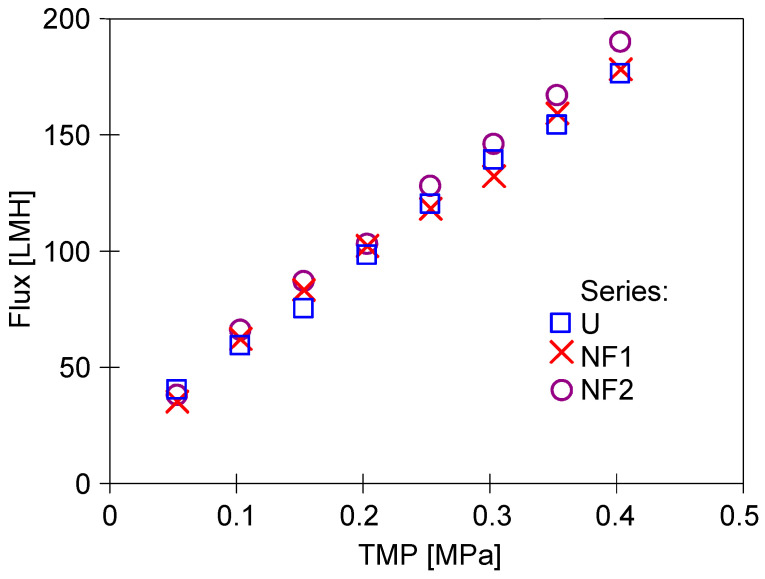
Changes in the permeate flux during membrane cleaning performed after UF of carwash wastewater. Points: U—installation washed with P3 Ultrasil 11, NF1, NF2—rinsing with NF permeate (2 h).

**Figure 10:**
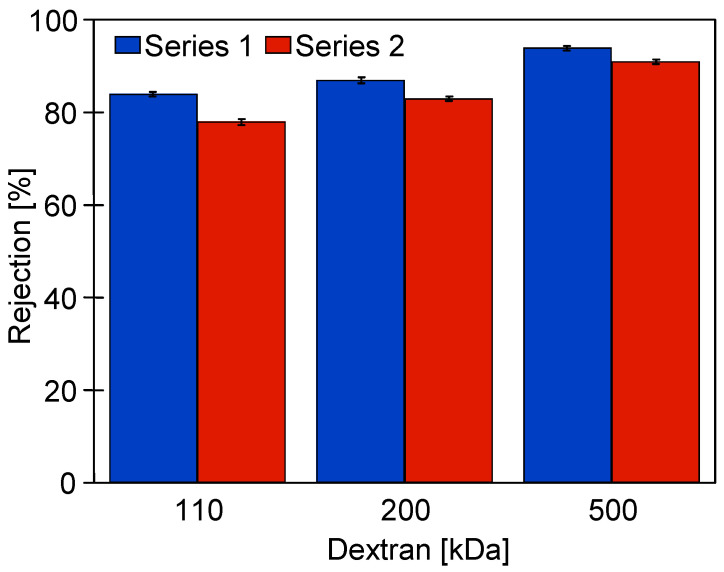
Dextran (1 g/L) rejection by the studied FP100 membranes. Series 1—initial period (Figure 6, Start), Series 2—after carwash wastewater separation (Figure 8) and chemical cleaning (Figure 7).

After completion of the effluent separation, the unit was rinsed with water and washed with P3 Ultrasil 11 solution. The permeate flux obtained after the chemical wash was similar to that obtained during the two additional rinses with NF permeate (Figure 9). It is worth noting that the flux values obtained are similar to those obtained after the stabilisation of the properties of the new membrane (Figure 4, series ‘S9’). According to the aforementioned data, the tested membranes achieved stable permeability properties as a result of the compression and chemical cleaning processes being repeated several times. Final dextran separation studies showed a slight decrease in the retention degree; e.g., the separation of 500 kDa dextran decreased from 94 to 91% (Figure 10). It can be concluded that repeated washing of the membranes with P3 Ultrasil 11 and preservation with sodium metabisulfite solution did not significantly affect the separation properties of FP100 membranes. The parameters obtained in this way can therefore be used to further investigate the suitability of the studied commercial module for the application of the UF process to the separation of car wash wastewater.

## 4. Conclusions

UF tests have confirmed that the performance of membranes in commercial modules can change significantly during the first few days of operation. Therefore, in industrial application studies, the results obtained should be related to rejection and initial permeate flux values stabilised for industrial process conditions.

The new UF membranes were compressed under process pressure, resulting in a reduction in permeate flux. For a given TMP value, the flux stabilised after 2–3 h of process; however, this was not a permanent state. Increasing or decreasing the TMP level caused the flux to stabilise again. Notably, relative stability in the operation of the UF module was achieved after 3 days of testing.

Fouling and chemical cleaning alter the separation properties of membranes. For this reason, the fouling–washing cycle should be repeated at least 2–3 times before studying the effect of these processes on the performance of the industrial plant in UF tests. Hence, it can be clearly indicated that it is important to use a feed whose components do not cause internal fouling/scaling at this stage and that the cleaning agents will restore the initial permeate flux.

## Figures and Tables

**Table 1:** Results of TOC analysis of dextran rejection by the studied membranes. Series D1—test for new membranes (after its stabilisation, Figure 3), Series D2—membranes after chemical washing. Water—feed composition before dextran addition to the feed tank. F—feed, P—permeate.

Dextran	IC [mg/L]		TOC [mg/L]		R_TOC_
[kDa]	F	P	F	P	[%]
Series D1					
water	2.41	1.73	0.58	0.48	-
40	2.65	2.11	19.02	6.11	67.9
70	2.73	2.15	20.20	6.69	66.9
110	2.70	2.19	19.57	5.34	72.7
200	2.72	2.18	20.10	4.88	75.7
Series D2					
water	3.40	-	0.65	-	-
110	3.29	2.75	32.41	9.95	69.3
water	3.04	-	0.38	-	-
200	2.98	2.43	17.56	5.37	69.4
water	2.97	-	0.64	-	-
500	3.36	2.86	16.90	3.96	76.6

## Data Availability

The original contributions presented in the study are included in the article; further inquiries can be directed to the corresponding author.
